# The circular RNA circ0005654 interacts with specificity protein 1 via microRNA-363 sequestration to promote gastric cancer progression

**DOI:** 10.1080/21655979.2021.1971031

**Published:** 2021-09-09

**Authors:** Cui Yang, Shengjin Han

**Affiliations:** a Department of Clinical Medicine, Wanxi Health Vocational College, Lu’an, Anhui, P.R. China; b Department of Emergency Surgery, Lu’an People's Hospital, Lu’an, Anhui, P.R. China

**Keywords:** Circ0005654, microRNA-363, sp1, myc, /β-catenin pathway, gastric cancer

## Abstract

Circular RNAs (circRNAs), a group of unique long noncoding RNAs, are involved in gastric carcinogenesis through multiple mechanisms, including interacting with microRNAs (miRNAs). Here, circ0005654, significantly upregulated in gastric cancer (GC), was chosen for further examination. circ0005654 was analyzed by RT-qPCR. The function of circ0005654 in GC cells was substantiated by loss-of-function assays. The mechanism of circ0005654 on miR-363/specificity protein 1 (sp1) axis was evaluated in GC cells by bioinformatics analysis, luciferase reporter, FISH, and ChIP assays. We observed that circ0005654 was enhanced in GC tissues and cells. Overexpression of circ0005654 was correlated with a poor long-term prognosis in patients with GC. Functionally, silencing of circ0005654 remarkably suppressed GC cell proliferation, migration and invasiveness *in vitro* and tumorigenesis and metastases *in vivo*. It was also established that circ0005654 served as a miR-363 sponge and enhanced sp1 expression. Furthermore, sp1 promoted GC carcinogenesis by regulating myc transcription to potentiate the Wnt/β-catenin pathway. In conclusion, circ0005654 expedites the GC development via miR-363/sp1/myc/Wnt/β-catenin axis and is a new biomarker for GC treatment regimen.

## Introduction

Gastric cancer (GC) holds accountable for more than 1,000,000 new diagnoses and a projected 783,000 deaths in 2018, leaving it the fifth most common malignancy and the third major cause of cancer-related mortality [1]. The majority of GC is closely linked to infectious agents, involving the bacterium Helicobacter pylori and Epstein-Barr virus [2]. Common risk factors for GC comprise of older age, male sex, tobacco consumption, radiation, as well as family history [3]. In spite of promoted incidence, GC patients in Asia have a better prognosis than those in the West, probably thanks to an active screening program and a more aggressive therapeutic measure [4]. Surgery remains the mainstay of treatment, and survival benefits were displayed by perioperative chemotherapy and postoperative chemoradiotherapy [5]. Consequently, studying molecular patterns and discovering novel treatments for GC are urgently needed, particularly for complicated gene mediation axes.

Circular RNAs (circRNAs) are a group of single-stranded RNAs that produce a covalently closed loop by joining the 3ʹ and 5ʹ ends, and the presence of circRNAs has been established for more than 2 decades [6]. The participation of circRNAs in different physiological process, including aging, insulin secretion in addition to tissue development has been underscored [7,8]. The competing endogenous RNA (ceRNA) hypothesis proposes that microRNAs (miRNAs) control the stability and transcription of mRNAs post-transcriptionally, which mainly involves mRNAs, transcribed pseudogenes and lncRNAs, and circRNAs have recently followed lncRNAs in being a research hotspot among the ceRNA family [9]. For instance, circREPS2 serves as a sponge of miR-558 to hinder GC progression by modulating RUNX3 [10]. In the current study, circ0005654 was identified as an upregulated circRNA in patients with GC. Moreover, miR-363 was revealed to be a target of circ0005654 with the help of StarBase (http://starbase.sysu.edu.cn/index.php) in GC. The tumor suppressive role of miR-363 has been evidenced in wide spectrum of cancers, including osteosarcoma [11], head and neck squamous cell carcinoma [12], colorectal cancer [13] as well as GC [14]. miR-363-3p was also reduced in hepatocellular carcinoma and suppressed tumorigenesis by targeting specificity protein 1 (sp1) [15], which was in line with our prediction. Moreover, circSCAF11 positively mediated sp1 expression via interacting with miR-421 in glioma [16]. Nevertheless, no study provided information on the importance of circ0005654 in GC. In this study, we speculated that circ0005654 may regulate GC cell proliferation, migration, invasion and apoptosis by interacting with sp1 via miR-363. The aim of the current study was to assess whether circ0005654 participated in the occurrence of GC and delved into the mechanism of action with the involvement of miR-363/sp1.

## Materials and methods

### Bioinformatics analysis

CircBase (http://circbase.org/) was utilized to find information about circ0005654, and UCSC (https://genome.ucsc.edu/) was applied to obtain the chromosomal location and host gene of circ0005654. The binding sites of sp1 to myc were predicted in ALGGEN (http://alggen.lsi.upc.es/). Moreover, StarBase (http://starbase.sysu.edu.cn/index.php), Targetscan (http://www.targetscan.org/), and miRanda (http://www.microrna.org/microrna/home.do) databases were utilized to predict potential target miRNA for circ0005654 and mRNA target of miR-363.

### Tissues

This study was permitted by the Ethics Committee of Lu’an People’s Hospital and was in compliance with the relevant guidelines. Informed consent was acquired from all participants. Fifty-seven pairs of GC and adjacent tissues (at least 3 cm away from the tumor lesion) were harvested between January 2015 and May 2017 from GC patients undergoing surgery in Lu’an People’s Hospital. All fresh samples were instantly preserved in RNA fixative reagent (BioTeke Corporation, Wuxi, Jiangsu, China) and preserved at −80°C until RNA was isolated. Patients were followed-up for three years after surgery, and their survival was recorded every three months.

### RT-qPCR

Extraction of RNA was performed using TRIzol (Thermo Fisher Scientific Inc., Waltham, MA, USA). NanoDrop 1000 spectrophotometer (Thermo Fisher) was utilized for protein concentration determination. The quality of extracted RNA was measured using an Agilent 2100 Bioanalyzer (Agilent Technologies, Santa Clara, CA, USA), and cDNA was synthesized by reverse transcription using a PrimeScript synthesis cDNA kit (Takara Biotechnology, Kyoto, Japan). After that, SYBR Green PCR Master Mix (Takara) was applied for qPCR. The primers utilized were as follows: circ0005654 (fwd, 5ʹ-GAGTTCATATCCGGAGCCACA-3ʹ; rvs, 5ʹ-CCTGTAGGCTTGATGCTGAAGA-3ʹ), miR-363 (fwd, 5ʹ- GCCGAGAATTGCACGGTAT-3ʹ; rvs, 5ʹ-CTCAACTGGTGTCGTGGA-3ʹ); sp1 (fwd, 5ʹ- GAGGTATTCGCACCAGAGGA-3ʹ; rvs, 5ʹ-ACCACCAGATCCATGAAGACC-3ʹ); myc (fwd, 5ʹ-TCTCAAGGCTCTGGAACTG-3ʹ; rvs, 5ʹ- GCCCCACACCCTGTGATG-3ʹ); glyceraldehyde-3-phosphate dehydrogenase (GAPDH, fwd, 5ʹ-GAAGGTGAAGGTCGGAGTC-3ʹ; rvs, 5ʹ- GAAGATGGTGATGGGATTTC-3ʹ) and U6 (fwd, 5ʹ- GCTCGCTTCGGCAGCACA-3ʹ; rvs, 5ʹ-GAGGTATTCGCACCAGAGGA-3ʹ). The 2^−ΔΔCt^ method was applied to calculate the relative expression with GAPDH or U6 small nuclear noncoding RNA (U6) as a loading control.

### Cell culture and treatment

Human GC cell lines MKN-45, HGC-27, NCI-N87, MKN-74 and the control cell line GES-1 were acquired from Institute of Biochemistry and Cell Biology (Shanghai, China). The species origin of the cell line was confirmed by PCR, and the cell lines were validated to be free of mycoplasma contamination using short tandem repeat analysis. The GC cells were grown in RPMI-1640 (Thermo Fisher) with 10% FBS (Gibco, Carlsbad, CA, USA) at 37°C with 5% CO_2._ GES-1 cells were grown in DMEM (Thermo Fisher) containing 10% FBS (Gibco). Lipofectamine 3000 (Thermo Fisher) was utilized as per to the manufacturer’s protocol. Small interfering (si) RNA targeting circ0005654 (si-circ0005654), circ0005654-overexpression (OE), miR-363 inhibitor, miR-363 mimic and sp1-OE were synthesized from Genepharma (Shanghai, China).

### Fluorescence in situ hybridization (FISH)

A FISH Tag RNA Green Kit long with Alexa Fluor™ 488 dye (Thermo Fisher) was used to determine the localization of circ0005654. The GC cells were seeded into 6-well culture plate and cultured to a confluence of about 80%. The GC cells were fixed with 4% paraformaldehyde at room temperature and cultured at 42°C for 60 min with 250 μL pre-hybridization solution, followed by an incubation with the fluorescent probe specific to circ0005654 overnight at 42°C. The nuclei were stained with 4ʹ,6-diamidino-2-phenylindole (Solarbio, Beijing, China) diluted with PBS/Tween for 5 min. After sealing with an anti-fluorescence quenching medium, five different fields of view were selected and photographed under a fluorescence microscope (Olympus Company, Tokyo, Japan).

### CCK-8 assay

CCK-8 assay was performed as described previously [17]. The cells were resuspended at 24 h post-transfection, seeded into 96-well plates (2 × 10^4^), and cultured for 96 h using CCK-8 (Beyotime, Shanghai, China). After 96 h, 10 µL CCK-8 solution was supplemented and incubated at 37°C for 120 min. The OD value of the wells was read at 450 nm using a microplate reader (Bio-Rad Laboratories, Hercules, CA, USA).

### Transwell assays

Matrigel (BD Biosciences, San Jose, CA, USA) was pre-applied to the bottom of the apical chamber for the invasion assay and allowed to stand at ambient temperature until solidified. A total of 1 × 10^5^ cells was supplemented into the apical chamber and cultured in serum-free medium. In addition, 500 µL medium plus 10% FBS as a chemoattractant was supplemented to the basolateral chamber. After being cultured at 37°C for 2 d, the cells retained in the apical chamber were removed, and the cells at the bottom of the basolateral chamber were fixed with 4% paraformaldehyde, stained with 0.5% crystal violet, and counted under the microscope (Olympus). For the migration assay, the cells were added into the apical chamber without Matrigel coating. The rest of the migration assay was carried out as described for invasion analysis.

### Hoechst33258 staining

Cell apoptosis was assessed as previously described [18]. The cells were resuspended with PBS and fixed in paraformaldehyde. The cells were washed with 0.01 mol/L PBS for 5 min, stained with Hoechst33258 working solution (Solarbio) for 15 min at ambient temperature, and sealed with the mixture of glycerin and PBS (1:9). The positive cells were viewed under a fluorescence microscope (Olympus), and the percentage of positive cells was calculated.

### Luciferase activity assay for the 3ʹuntranslated region (3ʹUTR) study

The wild type (WT) fragments of circ0005654 and sp1 3ʹUTR containing predicted binding site of miR-363 were from GenePharma (Shanghai, China). These fragments were cloned into the pmirGLO dual luciferase vectors (Promega Corporation, Madison, WI, USA), and the circ0005654/sp1-3ʹ-UTR-mutant (MT) plasmids containing the mutant miR-363 binding sites were generated using the QuikChange multipoint directed mutagenesis kit (Agilent). Subsequently, Lipofectamine 3000 transfection reagent (Thermo Fisher) was used to co-transfect miR-363 mimic or control with the corresponding vectors into HEK293T cells (ATCC). Firefly and Renilla luciferase activities were assessed using a dual-luciferase reporter gene assay system (Promega Corporation). Firefly luciferase activity was standardized to Renilla luciferase activity.

### Animal experiments

Twenty-five SPF female nude mice (4–6 weeks old, 20 ± 2 g, Vital River, Beijing, China) were allocated into control, miR-363 mimic, miR-363 mimic + negative control (NC), miR-363 mimic + circ0005654 and miR-363 mimic + sp1 groups (n = 5). A total of 4 × 10^6^ MKN-45 cells were dispersed in 2 mL saline and injected subcutaneously into each group of nude mice. Measurement of mouse tumor volume was performed at an interval of 7 days (tumor volume was calculated as L × W^2^/2, where L is the length and W is the width). After 28 d, the mice were euthanized by overdosed 1% sodium pentobarbital (120 mg/kg), and the tumors were weighed for histological experiments.

Twenty-five SPF female nude mice (4–6 weeks old, 20 ± 2 g, Vital River) were subjected to a tail vein injection of 4 × 10^6^ MKN-45 cells after different transfection (n = 5). After 45 days of injection, the mice were euthanized by injection of 1% sodium pentobarbital, and lung tissues were extracted and sectioned for paraffin embedding. Hematoxylin-eosin (HE) staining was performed using HE staining kit (Solarbio) to observe the metastases. The sections were dewaxed with xylene for 8 min, immersed in alcohol of gradient concentration, then reacted with hematoxylin for 15 min, differentiated in 1% hydrochloric acid alcohol for 0.5 min, and treated with eosin solution for 5 min, followed by dehydration, clearing and sealing. A microscope (Leica DM500) was utilized to observe the lung tissue sections, and five randomly selected fields of view were photographed. All animal experiments were permitted by the Animal Ethics Committee of Lu’an People’s Hospital.

### Chromatin immunoprecipitation (ChIP)

ChIP was conducted as previously described [19]. MKN-45 cells were fixed in 37% formaldehyde, collected in EP tubes and added with SDS lysis buffer and protease inhibitor complex (Thermo Fisher). ChIP was performed with the Pierce Agarose ChIP Kit (Thermo Fisher) after ultrasonic fragmentation. The samples were added with 900 μL ChIP dilution buffer and 60 μL proteinA agarose, and centrifuged at 1000 g for 1 min after mixing at 4°C for 1 h. The lysates were incubated either with antibodies specific for sp1 (1:30, ab231778, Abcam, Cambridge, MA, USA) or with control IgG (1:100, ab172730, Abcam). After incubation at 4°C overnight, 60 μL proteinA agarose and 250 μL elution buffer were added to each tube to wash the precipitate complexes. After a centrifugation for 15 min at ambient temperature, the supernatant was added with 20 μL NaCl (5 M) for decrosslinking. The recovered DNA fragments were dissolved in 100 μL ddH_2_O for PCR after re-elution.

### Western blot

Total protein was extracted from cells and tissues with the help of radioimmunoprecipitation assay buffer (Beyotime). The protein concentration was assessed using a bicinchoninic acid assay (Beyotime), and 30 µg protein was separated on a 10% SDS-polyacrylamide gel electrophoresis. The separated proteins were then transferred to NC membranes (Millipore Corp, Billerica, MA, USA) and sealed with tris-buffered saline containing 5% skim milk for 60 min at ambient temperature. The membranes were probed with monoclonal antibodies against sp1 (1:3000; ab124804, Abcam), β-catenin (1:8000; ab32572, Abcam), c-myc (1:1000; ab32072; Abcam) and GAPDH (1:2500, ab9485, Abcam) at 4°C overnight. The membranes were re-probed with HRP-labeled secondary antibodies (1:5000, ab205718, Abcam) for 60 min at ambient temperature. Protein bands were visualized using an enhanced chemiluminescent protein detection kit (Thermo Fisher), and signals were analyzed using Image J software (1.48, NIH, Bethesda, MD, USA).

### Data analysis

All data are illustrated as mean ± SD of at least three independent experiments. The results were analyzed by unpaired *t* test or one-way or two-way ANOVA followed by Tukey’s test with SPSS 22.0 (IBM SPSS Statistics, Chicago, IL, USA). The differences were deemed statistically significant with *p < 0.05.

## Results

Here, we aimed to explored the role of circ0005654 in GC. We conducted a series of *in vitro* and *in vivo* assays, and found that circ0005654 promoted GC progression and metastasis via regulating the miR-363/sp1/myc/Wnt/β-catenin axis. Therefore, we investigated the functional and clinical roles of circ0005654/miR-363/sp1 in GC, providing new insights into the pathogenesis of GC.

### Circ0005654 might be one of the prognostic markers for GC

**Figure 1.**
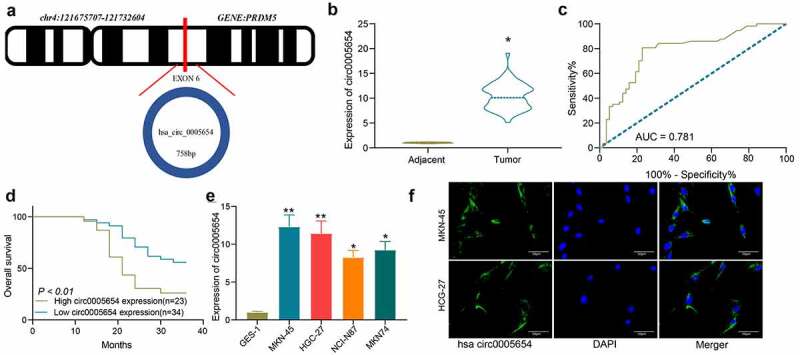
Circ0005654 is elevated in GC tissues and cells, which predicts a dismal survival. A, prediction of circ0005654 location, length, and host gene by circBase and UCSC websites; B, expression of circ0005654 in GC and adjacent tissues by RT-qPCR (*p < 0.05 by the paired *t* test); C, evaluation of the diagnostic effect of circ0005654 by a ROC curve using the Wilson/Brown analysis); D, survival analysis of patients with high or low expression of circ0005654 using the Kaplan-Meier analysis); E, circ0005654 expression in GC cells and GES-1 cells by RT-qPCR (*p < 0.05, **p < 0.01 by one-way ANOVA); F, the cellular localization of circ0005654 in MKN-45 and HGC-27 cells determined by FISH. Values are displayed as the mean ± SD on the basis of three independent experiments

Bioinformatics analysis showed that circ0005654 is chromosomally located at 121,675,707–121,732,604 bp on chromosome 4 and derived from the exon cyclization of its host gene, PRDM2 (Figure 1a). In order to determine the circ0005654 expression in GC, RT-qPCR was utilized to measure the circ0005654 expression in cancer and normal tissues from GC patients (n = 57). Quantification showed overexpression of circ0005654 in cancer tissues of GC patients (Figure 1b). The potential diagnostic value of circ0005654 was then assessed by ROC curve, which demonstrated that the area under the curve (AUC) was 0.781, and circ0005654 had a high diagnostic value for GC (Figure 1c). We analyzed the relationship between circ0005654 expression profile and the long-term prognosis of patients with gastrectomy, and found that patients with poor expression of circ0005654 had better prognoses (Figure 1d). Assessment of circ0005654 expression in GES-1 and GC cells (MKN-45, HGC-27, NCI-N87 and MKN-74) showed that circ0005654 was remarkably enhanced in GC cells (Figure 1e). As circ0005654 showed relatively high expression in MKN-45 cells and HGC-27 cells, we detected the location of circ0005654 in MKN-45 cells and HGC-27 cells by FISH and observed that circ0005654 was predominantly expressed in the cytoplasm (figure 1f). On the basis of the above results, we believed that circ0005654 is one of the prognostic markers for GC.

### Silencing of circ0005654 hinders GC cell activity

**Figure 2.**
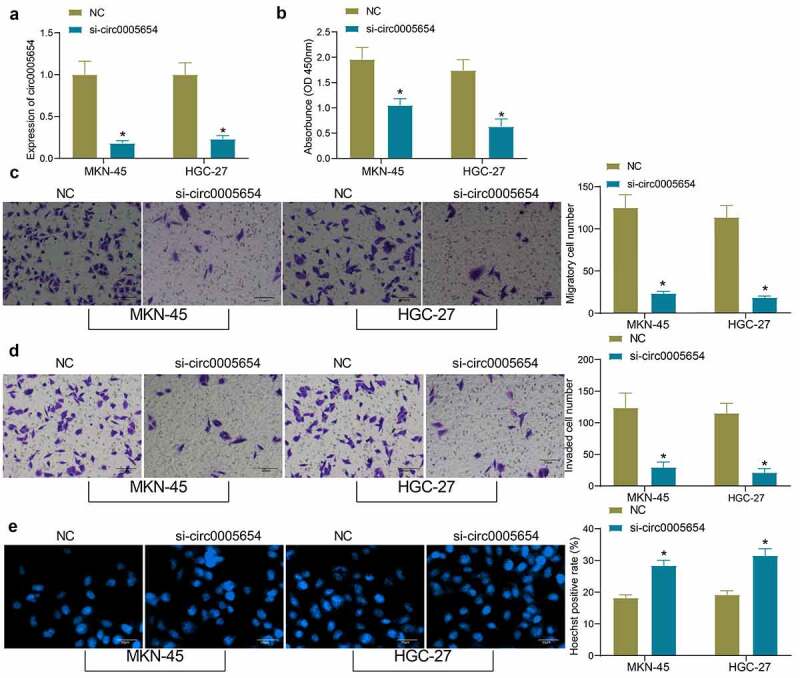
Circ0005654 suppresses the proliferation and aggressiveness of GC cells. A, circ0005654 expression after circ0005654 knockdown measured by RT-qPCR; B, OD values of cells after inhibition of circ0005654 assessed by CCK8; C, cell migration activity after inhibition of circ0005654 examined by Transwell assay; D, cell invasion activity after inhibition of circ0005654 examined by Transwell assay; E, cell apoptosis after inhibition of circ0005654 evaluated by Hoechst staining. Values are shown as the mean ± SD based on three independent experiments. *p < 0.05 *vs* cells transfected with NC. Experimental results were analyzed using two-way ANOVA followed by Tukey’s test

As circ0005654 is highly expressed in GC cells, we constructed GC cells with poor expression of circ0005654 and verified the cell transfection efficacy by RT-qPCR (Figure 2a). The proliferative activity of GC cells after knockdown of circ0005654 was measured by CCK8, and it was found that the inhibition of circ0005654 repressed the proliferative activity of GC cells (Figure 2b). After circ0005654 downregulation, the number of cells migrated and invaded into the basolateral chamber decreased drastically, and the cell migration and invasion were inhibited (Figure 2c, d). The cells were stained with Hoechst and observed under a fluorescence microscope to evaluate apoptosis. The number of Hoechst-positive cells increased significantly after circ0005654 downregulation, which indicated that inhibition of circ0005654 promoted the apoptosis of GC cells (Figure 2e). Through the above experiments, we demonstrated that circ0005654 knockdown reverts the malignant phenotype of GC cells.

### Circ0005654 regulates miR-363 and sp1 expression via the ceRNA mechanism

**Figure 3.**
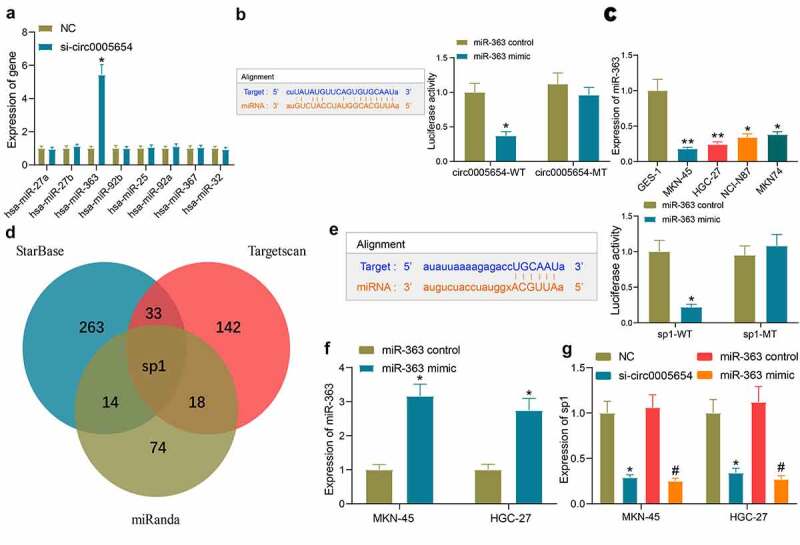
Circ0005654 functions as a miR-363 sponge to regulate sp1 in GC cells. A, expression of possible targeting miRNA of circ0005654 in GC cells examined by RT-qPCR; B, schematic representation of binding sites of miR-363 with circ0005654-WT or circ0005654-MT; C, miR-363 expression in GC cells and GES-1 cells by assessed by RT-qPCR; D, schematic illustration of the target mRNAs of miR-363 predicted by StarBase, Targetscan and miRanda; E, schematic representation of potential binding sites of miR-363 with sp1-WT or sp1-MT; F, the expression of miR-363 mimic in cells examined by RT-qPCR; G, sp1 expression in cells with poor expression of circ0005654 or high expression of miR-363. Values are shown as the mean ± SD based on three independent experiments. *#*p* < 0.05, **p < 0.01. Experimental results were analyzed using one-way (panel C) or two-way (panel A, B, E, F and G) ANOVA followed by Tukey’s test

Since circ0005654 is predominantly localized in the cytoplasm, we wondered that circ0005654 acts through a ceRNA mechanism. Upon searching for the downstream miRNA of circ0005654 by StarBase, miR-363 was revealed to be reduced in MKN-45 cells with the augment of circ0005654 by RT-qPCR screening (Figure 3a). The fluorescence intensity of the circ0005654-WT plasmid was significantly reduced by dual-luciferase assay in HEK293T cells co-transfected with the circ0005654 plasmids and miR-363 mimic, and binding relation between circ0005654 and miR-363 was validated (Figure 3b). We then assessed the expression of miR-363 in GC cells and GES-1 cells, and found that miR-363 expression was reduced in GC cells (Figure 3c). After identifying miR-363 as a target of circ0005654, we also predicted the target genes of miR-363 using StarBase, Targetscan, and miRanda. After screening out the genes by the Venn map, we observed that sp1 is a target of miR-363 (Figure 3d). So, after we verified the targeting relation between miR-363 and sp1 by dual-luciferase assay (Figure 3e), we transfected miR-363 mimic into GC cells (figure 3f) and tested the effect of circ0005654 downregulation and miR-363 upregulation on sp1 expression. It was found that circ0005654 downregulation and miR-363 upregulation both inhibited sp1 expression (Figure 3g). We then tentatively suggested that circ0005654 regulates sp1 expression through sponging miR-363.

### miR-363 expresses poorly and sp1 expresses highly in GC

**Figure 4.**
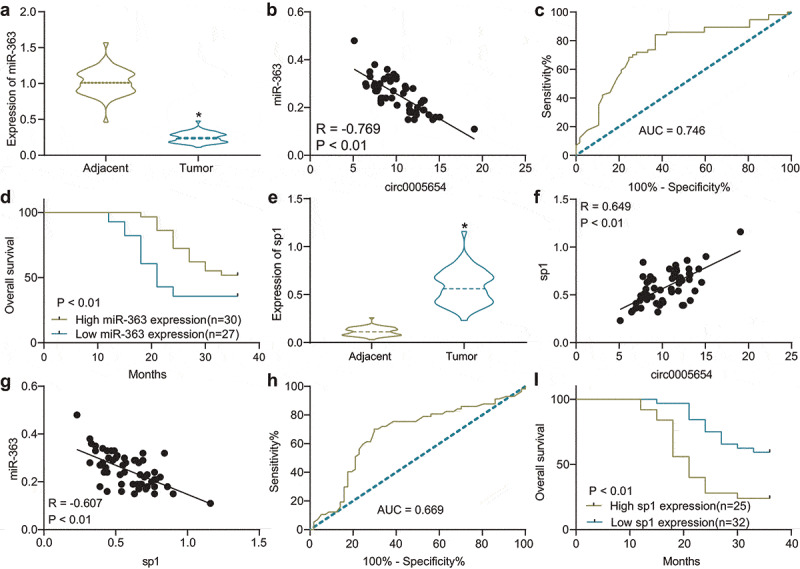
miR-363 and sp1 serve as diagnostic biomarkers in GC. A, miR-363 expression between GC and adjacent tissues assessed by RT-qPCR (*p < 0.05 by the paired *t* test); B, Person’s correlation analysis of circ0005654 and miR-363 expression in GC tissues; C, the diagnostic effect of miR-363 evaluated by ROC curves using the Wilson/Brown analysis; D, the survival curves of patients with poor or high expression of miR-363 using the Kaplan-Meier analysis; E, sp1 expression in GC and adjacent tissues assessed by RT-qPCR (*p < 0.05 by the paired *t* test); F, Person’s correlation analysis of circ0005654 and sp1 expression in GC tissues; G, Person’s correlation analysis of correlation between miR-363 and sp1; H, the predictive effect of sp1 on GC tissues evaluated by ROC curve using the Wilson/Brown analysis; I, the survival curves of patients with poor or high expression of sp1 using the Kaplan-Meier analysis

To detect the prognostic properties of miR-363 and sp1 in GC, the miR-363 expression in GC was revealed to be significantly poor relative to adjacent tissues (Figure 4a), and conversely correlated with the circ0005654 expression in GC tissues (Figure 4b). The diagnostic effect of miR-363 was found to be good with the ROC of 0.746 (Figure 4c). Analysis of the prognostic effect of miR-363 by survival curves revealed that the patients with high expression of miR-363 benefitted from a better prognosis and a higher survival time (Figure 4d). The expression of sp1 was significantly promoted in GC tissues (Figure 4e), and the correlation between sp1 and circ0005654 was positive (figure 4f). The sp1 expression in GC tissues was negatively correlated with miR-363 (Figure 4g), and detection of the diagnostic effect of sp1 revealed a larger area under the ROC (AUC = 0.669) (Figure 4h). Survival analysis of patients with high or low expression of sp1 revealed that sp1 high expression predicted a decreased survival (Figure 4i); these experiments suggest that both miR-363 and sp1 may be prognostic markers for GC.

### Circ0005654/miR-363/sp1 axis regulates GC cell malignant aggressiveness

**Figure 5.**
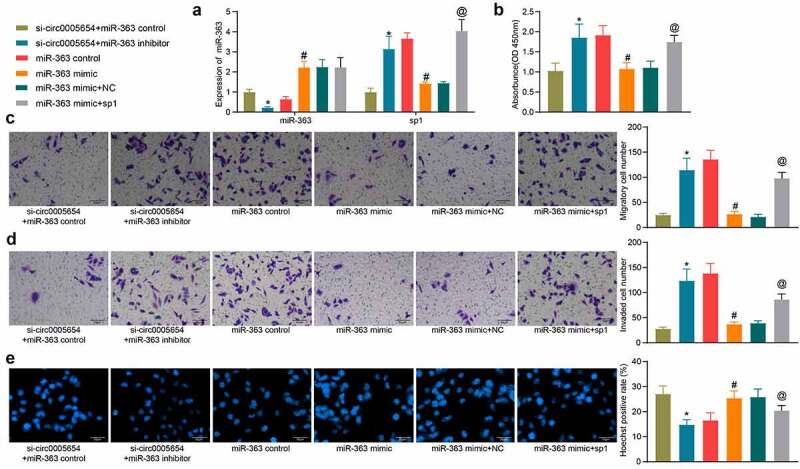
Circ0005654 or sp1 mitigates the effects of miR-363 on malignant phenotype in GC cells. A, circ0005654, sp1 and miR-363 expression after co-transfection measured by RT-qPCR; B, OD values of cells assessed by CCK8; C, cell migration activity examined by Transwell assay; D, cell invasion activity examined by Transwell assay; E, cell apoptosis evaluated by Hoechst staining. Values are shown as the mean ± SD based on three independent experiments. *p < 0.05 *vs* cells transfected with si-circ0005654 + miR-363 control; #*p* < 0.05 *vs* cells transfected with miR-363 control; @*p* < 0.05 *vs* cells transfected with miR-363 mimic + NC. Experimental results were analyzed using one-way (panel B, C, D, E) or two-way ANOVA (panel A) followed by Tukey’s test

To verify our above inferences about the relationships among circ0005654, miR-363, and sp1, we downregulated miR-363 expression in MKN-45 cells poorly expressing circ0005654 and upregulated sp1 in MKN-45 cells overexpressing miR-363, respectively. The success transfection was confirmed by RT-qPCR (Figure 5a). miR-363 inhibitor increased the OD value in the presence of si-circ0005654, while miR-363 mimic reduced cell proliferation ability, which was reversed by sp1 overexpression (Figure 5b). Similarly, the number of cells in the GC cell migration and invasion assay reduced by si-circ0005654 was flattened by miR-363 inhibitor. By contrast, sp1 overexpression mitigated the anti-migratory and anti-invasive effects of miR-363 mimic (Figure 5c, d). Hoechst staining showed that Hoechst positivity enhanced by si-circ0005654 was restored after miR-363 inhibitor, while upregulation of sp1 reversed the promoting effect of miR-363 mimic on apoptosis (Figure 5e). We verified the interrelationship between circ0005654/miR-363/sp1 and their effects on GC cell malignant aggressiveness.

### The circ0005654/miR-363/sp1 axis affects gastric carcinogenesis

**Figure 6.**
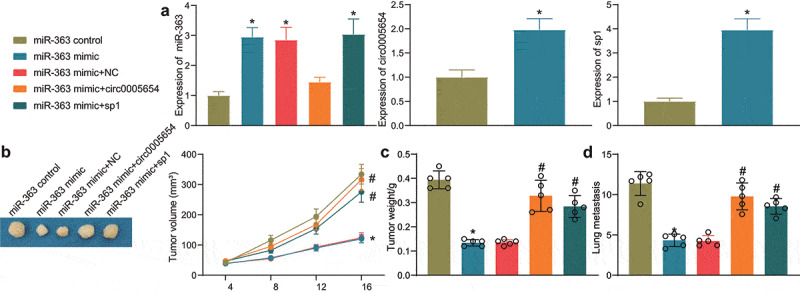
Circ0005654 or sp1 mitigates the effects of miR-363 on gastric carcinogenesis. A, circ0005654, sp1 and miR-363 expression in tissues using RT-qPCR to assess the efficiency of mouse model development; B-C, changes in the tumorigenic capacity of cells in xenograft models; D, HE staining for lung metastasis in mice after GC cell injection. *p < 0.05 *vs* mice injected with cells transfected with miR-363 control; #*p* < 0.05 *vs* mice injected with cells transfected with miR-363 mimic + NC. Experimental results were analyzed using one-way ANOVA followed by Tukey’s test

Mice were injected with cells overexpressing miR-363 mimic alone or with circ0005654/sp1 (Figure 6a) and observed the changes in tumor volume and weight. After 28 days, the tumor volume of the mice delivered with cells overexpressing miR-363 mimic was much smaller than that of the control group, while the overexpression of circ0005654 and sp1 inhibited the effect of miR-363 and elevated tumor volume (Figure 6b). After euthanasia, mouse tumors were isolated and weighed. miR-363 mimic contributed to a decline in tumor weight, while overexpression of circ0005654 and sp1 increased the tumor weight again (Figure 6c). Furthermore, we found that miR-363 reduced the number of lung metastatic nodules and the ability to metastasize in mice, while circ0005654 and sp1 increased the ability of cells to metastasize in mice, reversing the effect of miR-363 (Figure 6d). This series of results showed that circ0005654 and sp1 could reverse the action of miR-363 and thus lead to gastric carcinogenesis.

### Sp1 regulates the myc transcription-mediated Wnt/β-catenin pathway

**Figure 7.**
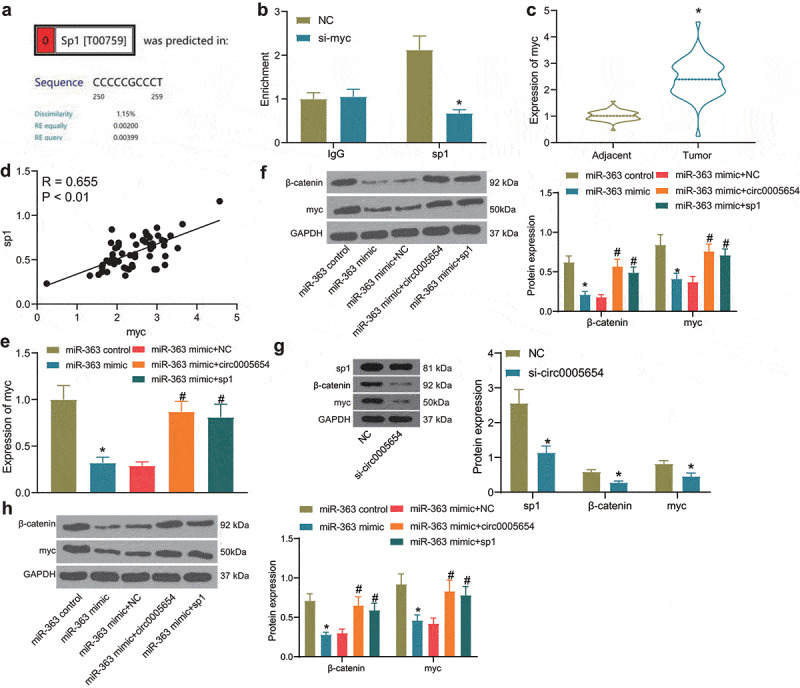
Sp1 binds to myc, thereby mediating the Wnt/β-catenin pathway. A, prediction of downstream genes and transcriptional sites of sp1 by bioinformatics analysis; B, the binding relation between myc and sp1 verified by ChIP experiments (*p < 0.05 by the two way ANOVA); C, myc expression between GC and adjacent tissues examined using RT-qPCR (*p < 0.05 by the paired *t* test); D, Person’s correlation analysis of sp1 and myc expression in GC tissues; E, expression of myc in MKN-45 cells after transfection examined using RT-qPCR (*#*p* < 0.05 by the one way ANOVA); F, the protein expression of myc and β-catenin in MKN-45 cells examined using western blot (*#*p* < 0.05 according to the two way ANOVA); G, the protein expression of sp1, myc and β-catenin in MKN-45 cells after circ0005654 knockdown examined using western blot (*#*p* < 0.05 according to the two way ANOVA); H, the protein expression of myc and β-catenin in xenografted mouse tumor tissues examined using western blot (*#*p* < 0.05 according to the two way ANOVA)

After validating the effects of the circ0005654/miR-363/sp1 axis in GC, we further searched for the sp1-mediated pathway. Since sp1 functions as a transcription factor in cells, we found that myc is one of the downstream factors of sp1 (Figure 7a) by bioinformatics analysis. As myc is a marker protein of the Wnt/β-catenin pathway, and Wnt/β-catenin pathway is an oncogenic signaling in GC, we speculated whether sp1 mediates Wnt/β-catenin pathway by regulating myc transcription. To test this hypothesis, ChIP was used to assess the enrichment of myc between −250 bp/-259 bp, it was found that knockdown of myc enriched sp1 protein in this region (Figure 7b). We then detected myc expression in GC and adjacent tissues and observed that myc was overexpressed in GC (Figure 7c) and positively correlated with sp1 (Figure 7d). Moreover, myc expression was increased after circ0005654 and sp1 upregulation and was decreased after miR-363 overexpression (Figure 7e). Finally, we tested the Wnt/β-catenin pathway activity in MKN-45 cells after transfection and found that Wnt/β-catenin pathway was impaired by miR-363 mimic, while elevated circ0005654 and sp1 inhibited the inhibitory effect of miR-363 on Wnt/β-catenin pathway inhibition (figure 7f). Also, we found a significant downregulation of sp1, myc, and β-catenin protein expression in MKN-45 cells after the downregulation of circ0005654 (Figure 7g). In tumor tissues from mice, we also found that overexpression of circ0005654 and sp1 was able to re-activate the Wnt/β-catenin pathway that was blocked due to overexpression of miR-363 (Figure 7h). This suggests that sp1 mediates Wnt/β-catenin pathway activity through the regulation of myc transcription.

## Discussion

In the current study, we expound the functions of circ0005654 in GC development. circ0005654 was overexpressed in GC tissues and cells. We also observed that circ0005654 upregulation predicted poor prognosis. In addition, circ0005654 knockdown hampered GC cell proliferation, migration and invasion, whereas stimulated apoptosis. Animal experiments suggested that circ0005654 silencing inhibited GC growth and metastases *in vivo*. We also observed that circ0005654 positively modulated sp1 through directly interacting with miR-363. sp1 initiated the transcription of myc, thereby mediating the Wnt/β-catenin pathway. In brief, our study established that circ0005654 is an oncogene via mediation of the miR-363/sp1/myc/Wnt/β-catenin axis in GC.

Large amounts of circRNAs have been recently identified to be aberrantly expressed in cancerous tissues, including GC. For instance, overexpression of circ-DONSON was proved in GC tissues and shared a positive correlation with advanced tumor, node, metastases stage and dismal prognosis [20]. Likewise, circPDSS1 might involve in GC tumorigenesis, and circPDSS1 expression contributed to an unsatisfactory prognosis in GC patients [21]. Herein, we introduced a newly found circRNA, circ0005654, and its regulatory axis to the system. The circ0005654 upregulation was found to possess high diagnostic values and could promote GC cell proliferation and aggressiveness, while inhibiting cell apoptosis. Depletion of circ0005654 exerted a great impact on curbing tumor growth and metastases.

In order to examine the molecular mechanism of circ0005654, we investigated the downstream signaling. Gene functional enrichment conducted by Pereira *et al*. indicated that circRNA/miRNA/mRNA axes have the potency to directly affect pathways that are well-known to be dysregulated in the event of gastric carcinogenesis [22]. Our results demonstrated that circ0005654 was mainly concentrated in the cytoplasm, and dual-luciferase reporter assay was performed to confirm that circ0005654 could competitively share miR-363 with sp1 mRNA, serving as a sponge. In the same vein, circ-sFMBT2 elevated the proliferation of GC cells via interacting with miR-182-5p to promote CREB1 expression [23]. Moreover, miR-363 was revealed to be involved in the ceRNA mechanism in GC by sponging lncRNA FEZF1-AS1 and lncRNA NNT-AS1 [24,25]. Also, miR-363 expression was reduced in esophageal squamous cell carcinoma samples and cells, which related to lymph node metastasis and tumor differentiation, and poor expression of miR-363 was acknowledged as an independent prognostic biomarker for esophageal squamous cell carcinoma [26]. In our study, miR-363 was observed to be downregulated in GC tissues and cells, which predicted a poor prognosis. As regards to the oncogenic role of sp1, highly expressed circ-ZNF609 sponged miR-150-5p to promote Sp1 expression, hence supporting the proliferation and metastatic abilities of nasopharyngeal carcinoma cells [27]. Our rescue experiments revealed that sp1 overexpression reversed the inhibitory role of miR-363 on cell viability, migration and invasiveness *in vitro* and metastases *in vivo*.

The transcriptional activator role of sp1 has been verified in hepatocellular carcinoma and glioma [28,29]. More relevantly, Sp1 has been recently reported to transcriptionally enhance the expression of CHAF1A in GC [30]. As a consequence, we assumed that sp1 exerted the same function as a transcriptional factor to modulate downstream pathway. Our bioinformatics prediction revealed that myc is a potential downstream effector of sp1. Intriguingly, lncRNA CRNDE was activated by sp1 to enhance osteosarcoma cell epithelial-mesenchymal transition via the Wnt/β-catenin signaling [31]. Kwon *et al*. described that cytochrome P450 1B1 elicited phenotypic modulation of MCF-7, MCF-10A, and MDA-MB-231 cells by activating the Wnt/β-catenin signaling through Sp1 induction [32]. Our data illustrated that the Wnt/β-catenin pathway was perturbed by miR-363 mimic in GC cells, while circ0005654 or sp1 overexpression abrogated the deficit of Wnt/β-catenin pathway. In brief, our findings propose that circ0005654 serves as a miR-363 sponge and elevates sp1, thereby affecting the transcription of myc and the subsequent Wnt/β-catenin pathway induction and potentiating GC proliferation, migration, invasion, and metastases.

## Conclusion

**Figure 8.**
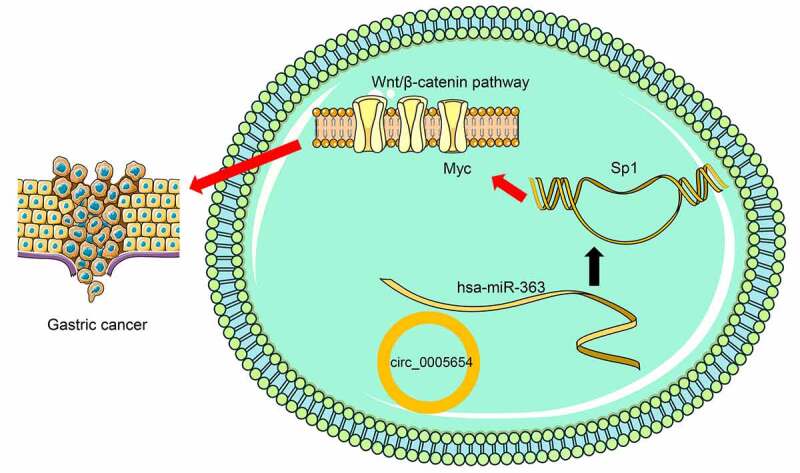
Summary of the regulation and mechanism in GC. circ0005654 positively regulated sp1 in GC by interacting with miR-363. sp1 activated the Wnt/β-catenin pathway by binding to myc

We concluded in our present study that overexpression of circ0005654 is significant in the tissues of GC patients and GC cell systems. Further experiments explained the central importance of silencing of circ0005654 in halting GC cell growth and migratory abilities (Figure 8), which may offer a new strategy for the diagnosis and treatment of GC. Since circ0005654 is a novel circRNAs, a more detailed understanding of the precise mechanisms underpinning circ0005654-mediated cancer development may expedite the development of new strategies for cancer treatment.

## Data Availability

All the data generated or analyzed during this study are included in this published article.

## Highlights

1.circ0005654 is overexpressed in GC.

2.circ0005654 promotes GC cell malignant phenotype.

3.circ0005654 binds to miR-363.

4.miR-363 regulates the expression of sp1.

5.sp1 mediates the Wnt/β-catenin signaling by promoting transcription of myc.
